# Case report: Intrathecal baclofen therapy improved gait pattern in a stroke patient with spastic dystonia

**DOI:** 10.3389/fneur.2024.1330811

**Published:** 2024-02-14

**Authors:** Kyung Min Kim, Tae Kwon Lee, Su Min Lee, Won Seok Chang, Su Ji Lee, Jihye Hwang, Sung-Rae Cho

**Affiliations:** ^1^Department and Research Institute of Rehabilitation Medicine, Yonsei University College of Medicine, Seoul, Republic of Korea; ^2^Department of Neurosurgery and Brain Research Institute, Yonsei University College of Medicine, Seoul, Republic of Korea; ^3^Graduate Program of Biomedical Engineering, Yonsei University College of Medicine, Seoul, Republic of Korea; ^4^Brain Korea 21 FOUR Project for Medical Science, Yonsei University College of Medicine, Seoul, Republic of Korea; ^5^Rehabilitation Institute of Neuromuscular Disease, Yonsei University College of Medicine, Seoul, Republic of Korea

**Keywords:** baclofen, spastic dystonia, intrathecal baclofen, gait analysis, stroke, muscle hypertonia

## Abstract

**Background:**

Intrathecal baclofen (ITB) therapy, a viable alternative for unsuitable candidates of conventional spasticity medications, is a preferred method of administration over the oral route. Owing to its enhanced bioavailability, ITB ensures a more effective delivery at the target site.

**Objective:**

There is a lack of conclusive evidence regarding the use of ITB treatment in managing ambulatory patients with spastic dystonia. Before ITB pump implantation, patients commonly undergo an ITB bolus injection trial to rule out potential adverse reactions and verify the therapeutic effects on hypertonic issues. In this report, we highlight a case of spastic dystonia, particularly focusing on an ambulatory patient who demonstrated significant improvement in both the modified Ashworth scale (MAS) score and gait pattern following the ITB injection trial.

**Case report:**

This case report outlines the medical history of a 67-year-old male diagnosed with left-side hemiplegia and spastic dystonia, resulting from his second episode of intracranial hemorrhage in the right thalamus. An ITB injection trial was initiated because the patient was not suitable for continued botulinum toxin injections and oral medications. This was due to the persistent occurrence of spastic dystonia in both the upper and lower extremities. The patient underwent a four-day ITB injection trial with progressively increasing doses, resulting in improved MAS scores and gait parameters, including cadence, step length, step time, stride length, and stride time were increased. Particularly, kinematic gait analysis demonstrates a substantial improvement of increased knee flexion in the swing phase in stiff knee gait pattern. These findings indicated a gradual reduction in spasticity-related symptoms, signifying the positive effect of the ITB injection trial. The patient eventually received an ITB pump implantation.

**Conclusion:**

In this post-stroke patient with spastic dystonia, ITB therapy has demonstrated effective and substantial management of spasticity, along with improvement in gait patterns.

## Introduction

Spasticity is defined as velocity-dependent hypertonia and tendon jerk hyperreflexia, resulting from stretch reflex excitability (1, 2). In contrast, dystonia involves involuntary muscle contractions, often leading to abnormal posturing and twisting movements (3). There are different types of dystonia, including focal dystonia such as blepharospasm, oromandibular dystonia, spasmodic dysphonia, torticollis, and writer’s cramp (4, 5). Generalized dystonia involves the leg, trunk, and at least one other body part (6). Spastic dystonia, which is a specific aspect of the upper motor neuron syndrome, is characterized by involuntary tonic contractions caused by the inability to relax the muscles (1). Spastic overactivity, a prevalent subtype of muscle hypertonia is frequently observed in patients with spinal or cerebral dysfunction. The term “spastic overactivity,” frequently employed to describe stretch-sensitive muscle overactivity, poses difficulties in precisely defining this intricate condition, as it includes spasms, involuntary motions, and undesired muscle activity (7).

The primary treatment for muscle overactivity and generalized dystonia typically involves oral medications, including baclofen, benzodiazepines, anticholinergics, antispasmodics, and levodopa (8, 9). Baclofen, an agonist for gamma-aminobutyric acid (GABA) B receptors, inhibits mono- and polysynaptic reflexes in the central and peripheral nervous systems, contributing to its therapeutic effect in reducing spasticity (10). Previous research explored the effectiveness of oral baclofen in managing poststroke spasticity, focusing on ankle stiffness and clonus during passive stretch, and identified factors influencing responsiveness (2). Nevertheless, oral baclofen may not always be effective for all patients due to the low concentration of baclofen in the cerebrospinal fluid (2). These non-responders exhibited a positive effect at higher cerebrospinal fluid levels achievable through intrathecal baclofen injection (2). Benzodiazepines demonstrate limited effectiveness, with approximately 16 to 23% showing a good clinical response (11). Trihexyphenidyl, a widely used agent, is a muscarinic acetylcholine receptor antagonist with a notably variable therapeutic dosage. While children may effectively respond to doses as low as 4 mg/day, adults are less likely to tolerate increasing dosages when necessary as they tend to be more sensitive to side effects such as memory loss, confusion, restlessness, insomnia, and nightmares (11). Intramuscular botulinum toxin injections can be beneficial in reducing focal spasticity in specific muscle groups (12, 13). Adverse effects, including dry mouth, fatigue, and flu-like symptoms, may occur when the drug spreads to unintended muscles and organs during botulinum toxin therapy (13). In some cases, these manifestations can escalate to systemic botulism signs. Although recent studies support the safety of botulinum toxin high-dose therapy, official documents recommend maximum botulinum toxin doses of onabotulinumtoxinA (Botox®, Allergan, Irvine, CA, USA) 400 units for upper limb or lower limb spasticity (14, 15). In the 1980s, intrathecal baclofen (ITB) therapy emerged as potentially effective next-in-line management for severe spasticity unresponsive to oral medications (9, 16–18), delivering concentrated drug doses directly into the intrathecal space to minimize systemic toxicity (19). Given the delicate nature of baclofen therapy and its potential complications, ITB bolus injections are administered to rule out adverse reactions and ensure the drug’s therapeutic effect on hypertonia before ITB pump implantation (9). ITB therapy offers reversible treatment of spasticity and effective modulation of muscle tone to reduce severe spasticity, for it was approved for the management of spasticity (20, 21). However, current literature highlights the necessity for additional research on the effectiveness of ITB treatment for mixed hypertonia, including spastic dystonia (21). In particular, additional research dedicated to ambulatory patient is essential.

Evaluating the effectiveness of treatment is crucial in managing spastic overactivity, and commonly employed methods include high-speed passive muscle stretching assessments like the modified Ashworth scale (MAS) and the modified Tardieu scale. However, these evaluations solely gauge resistance to passive movement and lack insight into muscle dynamics during dynamic tasks like gait (22). These scales also rely on subjective interpretation by clinicians, making it challenging to differentiate specific types of spastic hyperactivity, such as spasticity, spastic dystonia, and spastic co-contraction. Therefore, surface electromyography or gait analysis reflecting muscle activity during dynamic tasks has recently been used to classify spastic overactivity in detail (23, 24). Gait analysis, in particular, assesses muscle hyperactivity during functional tasks, allowing for a comprehensive understanding of specific movement patterns and continuous monitoring of various gait factors (23). Moreover, a transient decrease in spastic dystonia following ITB therapy can induce changes in gait pattern that are not easily discernible through casual observation. In such cases, gait analysis offers precise and objective measurements, detecting even subtle modifications in ambulatory performance (25).

Herein, we report an ambulatory patient with spastic dystonia demonstrating significant improvement in both the MAS score and gait pattern following the ITB bolus injection trial. The objective of this case study is to enhance comprehension of the response of adult spastic dystonia patients to ITB bolus injections and identify prospective recipients who may experience improved gait after ITB pump implantation. This study also represents the first stage in assessing whether the walking performance observed during the ITB bolus injection trial, evaluated through computerized gait analysis, is associated with changes following ITB pump implantation.

## Case description

We report the case of a 67-year-old male diagnosed with left-side hemiplegia with spastic dystonia resulting from an intracranial hemorrhage (Supplementary Figure S1). In 2004, the patient was initially admitted for facial paralysis and gait disturbance resulting from the initial episode of intracerebral hemorrhage in the right basal ganglia. The patient received conservative treatment and reported experiencing mild gait disturbance despite claiming no spasticity. Subsequently in 2012, a second episode of intracerebral hemorrhage occurred in the right thalamus, resulting in sensory loss upon placing his foot on the ground (Supplementary Figure S2). Despite undergoing traditional treatments, his dystonic movements persisted in both the left upper and lower limbs, with no significant improvement. He received botulinum toxin injections in specific muscles, such as the left shoulder flexor, elbow flexor, hip flexor, hip extensor, and ankle plantar flexor (onabotulinumtoxinA (Botox®, Allergan, Irvine, CA, USA) injection ranging from 50 to 100 units for each muscle, with a maximum dose of 300 units, distributed across two to four injection sites). He also received oral medications, namely Pregabalin (75 mg bid), Trihexyphenidyl (2 mg bid), Clonazepam (0.5 mg bid), and Baclofen (10 mg qid). In October 2022, the patient was eventually admitted undergoing ITB bolus injection trial and further evaluation for managing the symptoms.

Initial assessments included the modified Barthel Score (MBI) and Functional Independence Measure (FIM) to measure the level of independence in activities of daily living. Jebsen Hand Function Test (JHFT) was conducted to evaluate fine and gross motor hand function. Berg Balance Scale (BBS), a clinical test of a person’s static and dynamic balance abilities, was also administered. The severity of dystonia movements and their impact on daily life were evaluated using the Burke-Fahn-Marsden Dystonia Rating Scales (BFM-DRS). The results of these assessments, as presented in Table 1, indicated notable deficiencies in ambulation and stair climbing in activities of daily living. JHFT showed delayed fine and gross motor function on the left side. BBS indicated a lack of dynamic balance. BFM-DRS revealed dystonic movements in the left arm and leg, with evident walking disability.

**Table 1:** Changes in Modified Barthel Index (MBI), Functional Independence Measure (FIM), Jebsen Hand Function Test (JHFT), Berg Balance Scale (BBS), and Burke-Fahn-Marsden Dystonia Rating Scales (BFM-DRS) after intrathecal baclofen trial.

Measurements		Pre-ITB trial		Post-ITB trial	
MBI					
Personal hygiene		4		5	
Bathing self		3		3	
Feeding		10		10	
Toilet		8		8	
Stair climbing		8		8	
Dressing		10		10	
Bowel control		8		8	
Bladder control		10		10	
Ambulation		12		15	
Chair/bed transfer		15		15	
Total		88		92	
FIM					
Self-care	Eating	7		7	
	Grooming	5		6	
	Bathing	4		4	
	Dressing-upper	7		7	
	Dressing-lower	6		7	
	Toileting	4		4	
Sphincter control	Bladder	7		7	
	Bowel	7		7	
Transfers	Bed, Chair, W/C	6		7	
	Toilet	6		6	
	Tub, Shower	6		6	
Locomotion	Walk, Wheelchair	6		6	
	Stairs	4		5	
Comprehension	Comprehension	7		7	
	Expression	5		5	
Social cognition	Social interaction	7		7	
	Problem solving	7		7	
	Memory	7		7	
Total		108		112	
JHFT					
		Left	Right	Left	Right
Writing short sentence (/sec)		42.28	13.19	42.09	10.47
Card turning (/sec)		22.06	5.93	17.28	5.75
Picking up small common objects (/sec)		NT	10.19	NT	6.92
Simulated feeding (/sec)		41.06	10.07	23.02	6.69
Stacking checkers (/sec)		38.63	5.81	21.54	5.16
Moving light objects (/sec)		12.68	5.31	12.34	1.33
Moving heavy objects (/sec)		13.94	5.59	11.54	5.10
BBS					
Sitting to standing		3		4	
Standing unsupported		3		3	
Sitting unsupported		0		4	
Standing to sitting		4		4	
Transfers		2		3	
Standing with eyes closed		0		4	
Standing with feet together		1		4	
Reaching forward with outstretched arm		1		2	
Retrieving object from floor		1		2	
Turning to look behind		1		3	
Turning 360 degrees		1		1	
Placing alternate foot on stool		1		1	
Standing with one foot in front		1		2	
Standing on one foot		1		1	
Total		20		38	
BFM-DRS					
Dystonia movement scale	Eyes	0		0	
	Mouth	0		0	
	Speech/swallowing	0		0	
	Neck	0		0	
	Right arm	0		0	
	Left arm	4		4	
	Trunk	0		0	
	Right leg	0		0	
	Left leg	6		4	
	Total	10		8	
Disability scale	Speech	0		0	
	Writing	1		1	
	Feeding	0		0	
	Eating	0		0	
	Hygiene	2		2	
	Dressing	2		2	
	Walking	3		2	
	Total	8		7	

^*^W/C, wheelchair.

**Figure 1:**
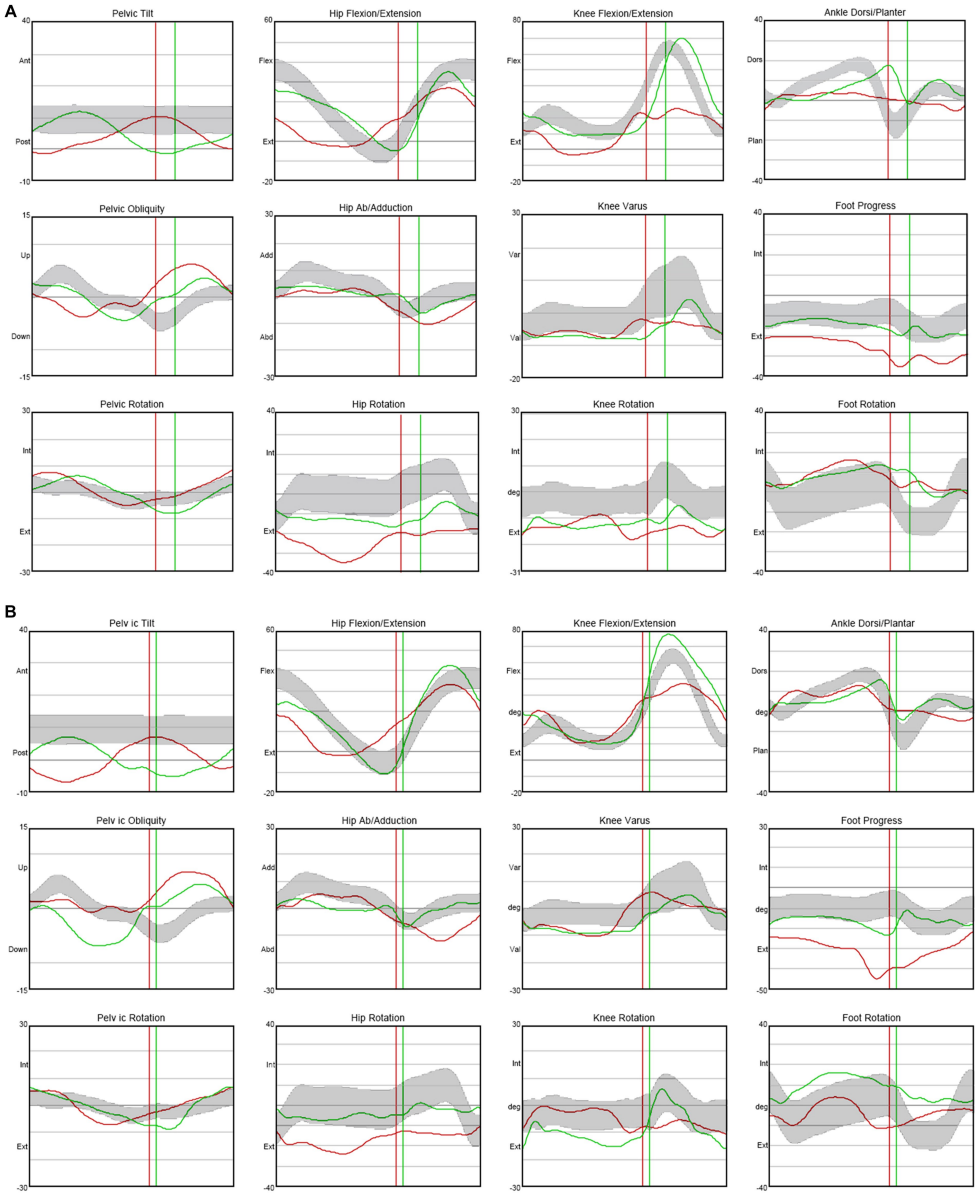
Kinematic changes in gait analysis after ITB injection trial. When compared with not injected state (Day 0) **(A)**, knee hyperextension was decreased and maximal dorsi flexion and peak hip extension were increased during the stance phase on the left side after 75 mcg injected state (Day 4) **(B)**. Moreover, maximal knee flexion was increased during the mid-swing phase on the left side. ITB, Intrathecal baclofen.

In terms of spasticity, the left upper and lower extremities exhibited MAS scores ranging from 1 to 2. Specifically, the knee flexor and extensor muscles were assigned a MAS score of 2 (Table 2). The results of pre-trial temporospatial gait parameters on the left side during gait analysis are detailed as follows: cadence of 88.1 steps/min, walking speed of 0.39 m/s, step length of 0.28 m, step time of 0.79 s, step width of 0.14 m, stride length of 0.54 m, and stride time of 1.36 s (Table 3). Based on kinematic analysis from the gait analysis illustrated in Figure 1A, no dissociated movement was observed between the pelvis and lower limbs in the pelvic area. In the hip region, there was reduced hip flexion observed at the initial heel contact on both sides, with more definite findings on the left side. Furthermore, a decrease in maximal hip flexion was noted during the swing phase, particularly on the left side. In the knee joint, there was knee hyperextension during the stance phase, accompanied by decreased knee flexion in the swing phase on the left side. The ankle area exhibited a fixed contracture at the neutral ankle angle, and there was a lack of ankle plantar flexion and dorsiflexion motion throughout the entire phase on the left side. These clinical observations suggested an involuntary, persistent tonic contraction preceding muscle stretch, indicative of spastic dystonia. Additionally, the patient displayed stiff knee gait pattern, which is recognized as one of the abnormal gait patterns.

**Table 2:** Changes in modified Ashworth scale after intrathecal baclofen injection trial.

Left muscle	Day 0	Day 1	Day 2	Day 3	Day 4
	0 mcg	12.5 mcg	25 mcg	50 mcg	75 mcg
Shoulder flexor	G1+	G1+	G1+	G1	G1
Shoulder extensor	G0	G0	G0	G0	G0
Elbow flexor	G2	G2	G2	G1+	G1
Elbow extensor	G1+	G1+	G1+	G1+	G1
Hip flexor	G1+	G1+	G1+	G1	G1
Hip extensor	G1+	G1+	G1+	G1	G1
Knee flexor	G2	G2	G1+	G1	G1
Knee extensor	G2	G2	G1+	G1	G1
Ankle dorsi flexor	G0	G0	G0	G0	G0
Ankle plantar flexor	G1	G1	G1	G1	G0

**Table 3:** Changes in temporospatial gait parameter after intrathecal baclofen injection trial.

Gait parameter	Day 0 (0 mcg)		Day 4 (75 mcg)	
	Left	Right	Left	Right
Cadence (steps/min)	88.1	92.0	95.2	92.0
Walking speed (m/s)	0.39	0.39	0.54	0.53
Step length (m)	0.28	0.23	0.43	0.25
Step time (s)	0.79	0.51	0.72	0.58
Step width (m)	0.14	0.13	0.16	0.14
Stride length (m)	0.54	0.51	0.68	0.69
Stride time (s)	1.36	1.30	1.26	1.30

Typically, the trial to assess the impact of ITB bolus injection at the L2 spinal level involves incremental doses administered over four consecutive days, ranging from 12.5 mcg to 75 mcg. No administration occurs on Day 0. The sequence comprises 12.5 mcg on Day 1, followed by 25 mcg on Day 2, 50 mcg on Day 3, and 75 mcg on Day 4 (Supplementary Figure S1).

The therapeutic impact of ITB on spastic dystonia was assessed through clinical evaluations, encompassing the MAS score and gait analysis. Kinematic changes during gait analysis were recorded for various parameters, including pelvic tilt, hip flexion/extension, knee flexion/extension, ankle dorsi/plantar, pelvic obliquity, hip abduction/adduction, knee varus, foot progress, pelvic rotation, hip rotation, knee rotation, and foot rotation (Figure 1). Serial assessments of the MAS score demonstrated a gradual reduction in symptoms associated with spastic dystonia with increasing doses of baclofen, suggesting a positive dose–response relationship (Table 2). Gait parameters, including walking velocity, and secondary temporospatial factors like cadence, step length, step time, stride length, and stride time, exhibited enhancements as well (Table 3). Moreover, kinematic analysis revealed significant improvement (Figure 1B). In the stance phase on the left side, there was a reduction in knee hyperextension, an increase in maximal dorsi flexion, and heightened peak hip extension. There was also a significant augmentation in maximal knee flexion observed during the mid-swing phase on the left side. These changes suggest enhanced gait stability and coordination. Follow-up evaluations, which included MBI, FIM, JHFT, BBS, and BFM-DRS demonstrated enhancements in overall activities of daily living, functional recovery in the left upper limb including the hand, improved dynamic balance, and a reduction in dystonic movements after the ITB injection trial (Table 1; Supplementary Figure S3). Observing positive effects in the ITB injection trial, the decision was made to proceed with ITB pump implantation. On December 13th, 2022, the patient underwent ITB pump implantation at the T3 spinal level during a neurosurgical hospitalization. After implantation, the patient had regular outpatient visits, and the baclofen dosage was adjusted based on symptom improvement and side effects. Spastic dystonia in the left upper and lower limbs improved, leading to a gradual increase in baclofen dosage from 50 mcg/day to 85 mcg/day. However, later, the patient complained of weakness in the right upper and lower limbs, resulting in a reduction to 75 mcg/day. As rehabilitation therapy was implemented concurrently, the dosage was gradually increased, and the patient currently maintains 105 mcg/day without experiencing weakness. Five months post-ITB pump implantation, the patient’s spasticity, the level of independence in activities of daily living, hand function and dynamic balance were assessed during a follow-up outpatient clinic visit to confirm the treatment effectiveness. In the follow-up assessment, the MAS score for the left upper and lower extremities remained at 1, the MBI total score was 90, and the BBS total score was 32. A comparison with Tables 1, 2; Supplementary Figure S3, and revealed that the overall improvements were consistently maintained compared to the pre-ITB trial.

## Discussion

We observed that ITB therapy resulted in a reduction of lower-extremity spastic hypertonia and improvement in gait function for a patient with hemiplegia and spastic dystonia following intracranial hemorrhage, ultimately leading to the decision for ITB pump implantation. The positive correlation between clinical spasticity reduction and functional improvement is highlighted in Table 1 and Supplementary Figure S3. Standard treatments for muscle overactivity and generalized dystonia, including oral medications and intramuscular botulinum toxin injections, may yield varied responses and side effects due to individual physiological differences (2, 8, 9, 12). Despite attempting oral medication and botulinum toxin muscle injections with no apparent symptom improvement, the patient sought a new therapeutic approach, leading to the consideration of ITB pump implantation. Although multiple prior studies propose the implantation of an intrathecal baclofen (ITB) pump as a therapeutic approach for spastic hypertonic conditions (9, 19, 26–28), there is a scarcity of research specifically addressing patients with spastic dystonia. The prevalence of spasticity and spastic dystonia is inadequately explored, with some studies indicating that spastic dystonia might be more common than spasticity in individuals who have experienced a stroke (24). To differentiate between spasticity and spastic dystonia, gait analysis or surface electromyography is frequently employed, as they offer insights into muscle activity during dynamic tasks (23, 24).

In this study, the patient exhibited spastic dystonia, as assessed through the MAS, BFM-DRS, and gait analysis. Dystonia movements and spasticity were observed in the left upper and lower extremities, with the elbow flexor, knee extensor, and knee flexor displaying more pronounced spasticity than other muscles (Tables 1, 2). Gait analysis revealed an overall abnormal gait pattern in this case (Figure 1A). Specifically, the left hip area showed decreased hip flexion in the stance phase and reduced maximal hip flexion in the swing phase. In the left knee area, knee hyperextension was noted in the stance phase, accompanied by decreased knee flexion in the swing phase. The left ankle area displayed a lack of ankle plantar and dorsi flexion motion throughout the entire gait cycle. These findings collectively indicated spasticity in the hip flexor, knee extensor, knee flexor, and ankle plantar flexor muscles. Furthermore, coactivation of knee extensor and knee flexor was identified. These results suggested stiff knee gait, a prevalent gait dysfunction in approximately 60% of stroke patients with gait disorders (29). Although the precise pathology of stiff knee gait is not fully elucidated, it may arise due to spasticity in the hip flexor, knee extensor, knee flexor, and ankle plantar flexor muscles, as well as coactivation of knee flexor and knee extensor muscles (30–32).

Throughout the ITB bolus injection trial, we continuously assessed spasticity using the MAS score and analyzed gait patterns through gait analysis. The MAS score consistently decreased, indicating a reduction in spasticity (Table 2). Temporospatial gait parameters, including cadence, step length, step time, stride length, and stride time, showed overall improvement (Table 3). Notably, gait velocity, a key measure for assessing functional response to ITB bolus injection trial (26), demonstrated improvement from 0.39 m/s to 0.54 m/s on the left side. Since velocity is the product of stride length and cadence, we examined the relation between these three parameters, and the improvements in stride length (from 0.54 m to 0.68 m) and cadence (from 88.1steps/min to 95.2 steps/min) were also observed. A significant increase in step length, indirectly reflecting gait balance, was also observed, improving from 0.28 m to 0.43 m. The decreased peak of hip extension, knee hyperextension, and decreased maximal dorsi flexion are related to spasticity of the knee extensor, plantar flexor muscles, and coactivation of knee extensor and knee flexor muscles, indicating stiff knee gait pattern (Figure 1A). According to kinematic analysis, knee hyperextension was decreased, and maximal dorsi flexion and peak hip extension were increased during the stance phase on the left side. Maximal knee flexion in particular showed a significant increased during the mid-swing phase on the left side (Figure 1B). Consequently, stiff knee gait pattern was improved after the ITB bolus injection.

Considering previous indications for ITB pump implantation, it is necessary to consider ITB pump insertion in patients with severe spasticity, especially indicated by MAS score of 3 or higher in the lower limbs, that is not relieved by oral muscle relaxants and adjuvant therapy including botulinum toxin injections and exercise (33–35). Individualized objectives may be assigned to each patient; patients capable of mobility may aim to enhance their movement, while those less capable of mobility may require attention to nursing care or ensuring comfortable sitting arrangements (36). Despite having a hemiplegic sequela, this patient could walk with some limitation and his level of spasticity was not severe (the MAS score 1 to 2). However, to improve gait function, an ITB bolus injection trial was attempted before considering the ITB pump insertion. Throughout the ITB bolus injection trial, the patient encountered a decrease in spasticity along with improvements in gait parameters and patterns. We expect that this case report will serve as evidence for the need to actively consider inserting an ITB pump according to each patient’s goals, even in patients with symptoms of spastic dystonia.

Limitations in this study include the fact that the patient underwent an ITB bolus injection trial after a considerable duration from the onset of symptoms, suggesting that spastic hypertonia may not have been the sole factor influencing his gait function. Other post-stroke sequelae, such as joint contractures and muscle shortening, could have contributed to abnormal gait patterns. Analyzing cases that undergo ITB bolus injection trials and ITB pump implantation as soon as possible after the onset of symptoms may provide more effective insights into efficacy. In this study, the efficacy of spastic dystonia treatment was validated through a four-day clinical trial utilizing gait analysis. Follow-up outpatient visits five months post-ITB pump implantation revealed that the patient’s condition remained improved, as indicated by the MAS, MBI, JFHT, and BBS. These outcomes highlight the necessity for additional long-term follow-up studies to substantiate the therapeutic benefits of ITB therapy for spastic dystonia via gait analysis. Large-scale clinical studies are also necessary to validate these findings and explore the long-term benefits and safety of ITB therapy for spastic dystonia. As more evidence accumulates, ITB therapy may emerge as an essential and effective treatment option for this complex and challenging movement disorder.

## Conclusion

The case report presented herein provides insights into the potential efficacy of ITB therapy for managing spastic dystonia in patients with stroke. The significant improvement in gait pattern and the MAS score following the ITB bolus injection trial suggested that ITB treatment may be a viable treatment option for spastic dystonia cases that do not respond to conventional therapies.

## Data availability statement

The original contributions presented in the study are included in the article/Supplementary material, further inquiries can be directed to the corresponding authors.

## Ethics statement

Ethical approval was not required for the study involving humans in accordance with the local legislation and institutional requirements. Written informed consent to participate in this study was not required from the participants or the participants’ legal guardians/next of kin in accordance with the national legislation and the institutional requirements. Written informed consent was obtained from the individual(s) for the publication of any potentially identifiable images or data included in this article.

## Author contributions

KK: Conceptualization, Data curation, Formal analysis, Investigation, Methodology, Project administration, Validation, Visualization, Writing – original draft, Writing – review & editing. TL: Data curation, Investigation, Methodology, Writing – review & editing. SML: Conceptualization, Data curation, Investigation, Writing – review & editing. WC: Conceptualization, Project administration, Validation, Writing – review & editing. SL: Formal analysis, Methodology, Writing – review & editing. JH: Conceptualization, Project administration, Supervision, Validation, Writing – original draft, Writing – review & editing, Formal analysis. S-RC: Conceptualization, Formal analysis, Investigation, Project administration, Supervision, Validation, Writing – review & editing.

## References

[1] Marinelli L Curra A Trompetto C Capello E Serrati C Fattapposta F. Spasticity and spastic dystonia: the two faces of velocity-dependent hypertonia. J Electromyogr Kinesiol. (2017) 37:84–9. doi: 10.1016/j.jelekin.2017.09.005, PMID: 28985544

[2] Mizuno S Takeda K Maeshima S Shigeru S. Effect of oral baclofen on spasticity poststroke: responders versus non-responders. Top Stroke Rehabil. (2018) 25:438–44. doi: 10.1080/10749357.2018.1474422, PMID: 29768106

[3] Roman Casul YA Humbert ML Farooqui A Wagle Shukla A Nagaraja N. Dystonia as a presenting feature of acute ischemic stroke: a case report and literature review. Cureus. (2021) 13:e17272. doi: 10.7759/cureus.17272, PMID: 34540493 PMC8448260

[4] Nutt JG Muenter MD Aronson A Kurland LT Melton LJ 3rd. Epidemiology of focal and generalized dystonia in Rochester, Minnesota. Mov Disord. (1988) 3:188–94. doi: 10.1002/mds.870030302, PMID: 3264051

[5] Jinnah HA Berardelli A Comella C Defazio G Delong MR Factor S. The focal dystonias: current views and challenges for future research. Mov Disord. (2013) 28:926–43. doi: 10.1002/mds.25567, PMID: 23893450 PMC3733486

[6] LeDoux MS. Dystonia: phenomenology. Parkinsonism Relat Disord. (2012) 18:S162–4. doi: 10.1016/S1353-8020(11)70050-5 22166421 PMC4869992

[7] Gracies JM. Pathophysiology of spastic paresis. II: emergence of muscle overactivity. Muscle Nerve. (2005) 31:552–71. doi: 10.1002/mus.20285, PMID: 15714511

[8] Bethoux F. Spasticity management after stroke. Phys Med Rehabil Clin N Am. (2015) 26:625–39. doi: 10.1016/j.pmr.2015.07.003 26522902

[9] Albright AL Barry MJ Shafton DH Ferson SS. Intrathecal baclofen for generalized dystonia. Dev Med Child Neurol. (2001) 43:652–7. doi: 10.1017/S0012162201001190 11665821

[10] Davidoff RA. Antispasticity drugs: mechanisms of action. Ann Neurol. (1985) 17:107–16. doi: 10.1002/ana.410170202, PMID: 2858176

[11] Cloud LJ Jinnah HA. Treatment strategies for dystonia. Expert Opin Pharmacother. (2010) 11:5–15. doi: 10.1517/14656560903426171, PMID: 20001425 PMC3495548

[12] Santamato A Cinone N Panza F Letizia S Santoro L Lozupone M. Botulinum toxin type a for the treatment of lower limb spasticity after stroke. Drugs. (2019) 79:143–60. doi: 10.1007/s40265-018-1042-z 30623347

[13] Floresta G Patamia V Gentile D Molteni F Santamato A Rescifina A. Repurposing of FDA-approved drugs for treating iatrogenic botulism: a paired 3D-QSAR/docking approach(dagger). ChemMedChem. (2020) 15:256–62. doi: 10.1002/cmdc.201900594, PMID: 31774239

[14] Dressler D Altavista MC Altenmueller E Bhidayasiri R Bohlega S Chana P. Consensus guidelines for botulinum toxin therapy: general algorithms and dosing tables for dystonia and spasticity. J Neural Transm (Vienna). (2021) 128:321–35. doi: 10.1007/s00702-021-02312-4, PMID: 33635442 PMC7969540

[15] Field M Splevins A Picaut P van der Schans M Langenberg J Noort D. AbobotulinumtoxinA (Dysport((R))), OnabotulinumtoxinA (Botox((R))), and IncobotulinumtoxinA (Xeomin((R))) neurotoxin content and potential implications for duration of response in patients. Toxins (Basel). (2018) 10:535. doi: 10.3390/toxins10120535, PMID: 30551641 PMC6316182

[16] Ertzgaard P Campo C Calabrese A. Efficacy and safety of oral baclofen in the management of spasticity: a rationale for intrathecal baclofen. J Rehabil Med. (2017) 49:193–203. doi: 10.2340/16501977-2211, PMID: 28233010

[17] Dykstra D Stuckey M DesLauriers L Chappuis D Krach L. Intrathecal baclofen in the treatment of spasticity. Acta Neurochir Suppl. (2007) 97:163–71. doi: 10.1007/978-3-211-33079-1_2 PMID: 17691372

[18] Taira T Hori T. Intrathecal baclofen in the treatment of post-stroke central pain, dystonia, and persistent vegetative state. Acta Neurochir Suppl. (2007) 97:227–9. doi: 10.1007/978-3-211-33079-1_31 PMID: 17691381

[19] Winter G Beni-Adani L Ben-Pazi H. Intrathecal baclofen therapy-practical approach: clinical benefits and complication management. J Child Neurol. (2018) 33:734–41. doi: 10.1177/0883073818785074, PMID: 30009656

[20] Penn RD Kroin JS. Continuous intrathecal baclofen for severe spasticity. Lancet. (1985) 2:125–7. PMID: 2862320 10.1016/s0140-6736(85)90228-4

[21] Abdelmageed S Horak VJ Mossner J Wang R Krater T Raskin JS. Safety and efficacy of intrathecal baclofen trials for the treatment of hypertonia: a retrospective cohort study. J Neurosurg Pediatr. (2023):1–6. doi: 10.3171/2023.11.PEDS23473, PMID: 38064708

[22] Biering-Sorensen F Nielsen JB Klinge K. Spasticity-assessment: a review. Spinal Cord. (2006) 44:708–22. doi: 10.1038/sj.sc.3101928, PMID: 16636687

[23] Roche N Bonnyaud C Reynaud V Bensmail D Pradon D Esquenazi A. Motion analysis for the evaluation of muscle overactivity: a point of view. Ann Phys Rehabil Med. (2019) 62:442–52. doi: 10.1016/j.rehab.2019.06.004, PMID: 31276837

[24] Trompetto C Curra A Puce L Mori L Serrati C Fattapposta F. Spastic dystonia in stroke subjects: prevalence and features of the neglected phenomenon of the upper motor neuron syndrome. Clin Neurophysiol. (2019) 130:521–7. doi: 10.1016/j.clinph.2019.01.012, PMID: 30776732

[25] Kadaba MP Ramakrishnan HK Wootten ME Gainey J Gorton G Cochran GV. Repeatability of kinematic, kinetic, and electromyographic data in normal adult gait. J Orthop Res. (1989) 7:849–60. doi: 10.1002/jor.1100070611, PMID: 2795325

[26] Horn TS Yablon SA Stokic DS. Effect of intrathecal baclofen bolus injection on temporospatial gait characteristics in patients with acquired brain injury. Arch Phys Med Rehabil. (2005) 86:1127–33. doi: 10.1016/j.apmr.2004.11.013, PMID: 15954050

[27] Nahm NJ Graham HK Gormley ME Jr Georgiadis AG. Management of hypertonia in cerebral palsy. Curr Opin Pediatr. (2018) 30:57–64. doi: 10.1097/MOP.0000000000000567 29135566

[28] Ben Smail D Peskine A Roche N Mailhan L Thiebaut I Bussel B. Intrathecal baclofen for treatment of spasticity of multiple sclerosis patients. Mult Scler. (2006) 12:101–3. doi: 10.1191/1352458506ms1232sr 16459726

[29] De Quervain IA Simon SR Leurgans S Pease WS McAllister D. Gait pattern in the early recovery period after stroke. J Bone Joint Surg Am. (1996) 78:1506–14. doi: 10.2106/00004623-199610000-00008, PMID: 8876578

[30] Piazza SJ Delp SL. The influence of muscles on knee flexion during the swing phase of gait. J Biomech. (1996) 29:723–33. doi: 10.1016/0021-9290(95)00144-1 9147969

[31] Akbas T Kim K Doyle K Manella K Lee R Spicer P. Rectus femoris hyperreflexia contributes to stiff-knee gait after stroke. J Neuroeng Rehabil. (2020) 17:117. doi: 10.1186/s12984-020-00724-z, PMID: 32843057 PMC7448457

[32] Kerrigan DC Roth RS Riley PO. The modelling of adult spastic paretic stiff-legged gait swing period based on actual kinematic data. Gait Posture. (1998) 7:117–24. doi: 10.1016/S0966-6362(97)00040-4, PMID: 10200381

[33] Chan DY Chan SS Chan EK Ng AY Ying AC Li AC. Blessing or burden? Long-term maintenance, complications and clinical outcome of intrathecal baclofen pumps. Surg Pract. (2018) 22:105–10. doi: 10.1111/1744-1633.12308, PMID: 30147745 PMC6099513

[34] Gilmartin R Bruce D Storrs BB Abbott R Krach L Ward J. Intrathecal baclofen for management of spastic cerebral palsy: multicenter trial. J Child Neurol. (2000) 15:71–7. doi: 10.1177/088307380001500201, PMID: 10695888

[35] Yoon YK Lee KC Cho HE Chae M Chang JW Chang WS. Outcomes of intrathecal baclofen therapy in patients with cerebral palsy and acquired brain injury. Medicine (Baltimore). (2017) 96:e7472. doi: 10.1097/MD.0000000000007472, PMID: 28834868 PMC5571990

[36] Dones I. Intrathecal baclofen for the treatment of spasticity. Acta Neurochir Suppl. (2007) 97:185–8. doi: 10.1007/978-3-211-33079-1_25 PMID: 17691375

